# Higher Levels of Autoantibodies Targeting Mutated Citrullinated Vimentin in Patients with Psoriatic Arthritis Than in Patients with Psoriasis Vulgaris

**DOI:** 10.1155/2013/474028

**Published:** 2013-03-18

**Authors:** Szandra Dalmády, Mária Kiss, László Képíró, László Kovács, Gábor Sonkodi, Lajos Kemény, Rolland Gyulai

**Affiliations:** ^1^Department of Dermatology and Allergology, University of Szeged, 6 Korányi Fasor, Szeged 6720, Hungary; ^2^Department of Rheumatology, University of Szeged, 57 Kálvária Sugárút, Szeged 6725, Hungary; ^3^Erzsébet Hospital, Hódmezővásárhely, Rheumatology and Rehabilitation Center of Kakasszék, 143 IV. Kerület, Székkutas 6821, Hungary; ^4^Dermatological Research Group of the Hungarian Academy of Sciences, University of Szeged, 6 Korányi Fasor, Szeged 6720, Hungary

## Abstract

Antibodies against citrullinated proteins/peptides (ACPAs), and especially antibodies targeting mutated citrullinated vimentin (anti-MCVs), are novel biomarkers of rheumatoid arthritis (RA). Whereas ACPAs are specific and sensitive markers for RA, there have hardly been any reports relating to ACPAs in psoriatic arthritis (PsA) or in psoriasis without joint symptoms (PsO). The aim of the present study was to investigate the prevalence of anti-MCVs in PsA and PsO. Serum anti-MCV titers were measured in 46 PsA and 42 PsO patients and in 40 healthy controls by means of a commercial enzyme-linked immunosorbent assay. The potential correlations of the serum autoantibody levels with several clinical and laboratory parameters were examined. The anti-MCV levels in the PsA patients were significantly higher than those in the PsO group. Among the clinical variables, the presence of tender knee joints and nail psoriasis was significantly associated with anti-MCV positivity in the PsA patients. Higher anti-MCV titers in the PsO patients were associated with a more severe disease course and with the early onset of psoriatic skin symptoms. Our results suggest that anti-MCVs can be used as novel markers in the diagnosis of PsA and in a subset of PsO patients.

## 1. Introduction

Antibodies targeting mutated citrullinated vimentin (anti-MCVs) belong in the group of anti-citrullinated protein/peptide antibodies (ACPAs). Antibodies against citrullinated cyclic peptides (anti-CCPs) are the most widely used members of the ACPA group [1–3]. The detection of ACPAs is a specific and sensitive marker for the diagnosis of rheumatoid arthritis (RA) [4–9]. The ACPAs are also of prognostic relevance. ACPA positivity is associated with a faster progression and a poorer outcome in RA [10–13]. Anti-MCVs and VCP2 (a peptide corresponding to the modified Epstein-Barr virus encoded protein 2 (EBNA-2)) are highly sensitive members of the ACPA group [14–16]. The anti-MCVs were recently reported to have higher diagnostic sensitivity than anti-CCPs and rheumatoid factor in RA [17–19], though conflicting results were found in another recent study as concerns the superiority of anti-MCVs over anti-CCPs in the diagnosis of RA [16]. Anti-MCVs are detectable in early RA patients, even before the symptoms are manifest, and are therefore presumed to be of prognostic value. Several recent studies have suggested that the production of these autoantibodies is associated with a faster disease progression and may well serve as a useful predictivemarkerof severe joint damage [20, 21]. Anti-MCVs target citrullinated vimentin. Vimentin, the main cytoskeletal component of the mesenchymal cells [22, 23], is not coded by DNA and can only be expressed by posttranslational modification, that is, enzymatic citrullination of the amino acid arginine. Vimentin contains 43 arginine residues, and the citrullination is catalyzed by the enzyme peptidylarginine deiminase found in monocytes and macrophages. Tissue inflammation and cell apoptosis lead to changes in the structure of the protein by enzymatic citrullination and activate the immune system by the increased production of autoantibodies [24]. Recent studies suggest that the enzymatic citrullination and the production of ACPAs may also be associated with other inflammative arthritis-associated autoimmune diseases [25–27].

Psoriatic arthritis (PsA) is a seronegative spondyloarthropathy that develops in up to 30 per cent of patients with psoriasis (National Psoriasis Foundation, http://www.psoriasis.org/). PsA occurs more frequently in subject with the HLA-B27 haplotype [28–30]. PsA has several different clinical phenotypes: oligoarticular, polyarticular, symmetrical, and asymmetrical peripheral joint inflammation or axial involvement [31, 32]. Various systems and criteria have been proposed to aid the diagnosis and classification of PsA [29, 33–37]. Although none of them are accepted unequivocally, the classification criteria described by Moll and Wright [37] and more recently the classification criteria for PsA (CASPAR) have been used most frequently [36]. The wide spectrum of disease expression often makes it difficult to distinguish PsA from RA or other spondyloarthropathies. Currently, there is no specific test that could be used reliably for the diagnosis of PsA. Moreover, a biomarker (or biomarkers) that could distinguish between different clinical phenotypes of PsA or between PsA and psoriasis vulgaris (PsO), or that could be used as a predictive marker for future PsA development in PsO patients, is still lacking.

Because of the several clinical similarities between PsA and RA, and in view of the fact that the anti-MCVs are highly sensitive markers in RA, we set out to investigate the prevalence of anti-MCVs in PsA and PsO patients. Possible associations between the anti-MCV titers and the clinical, and laboratory variables of PsA and PsO patients were also studied.

## 2. Materials and Methods

### 2.1. Study Population

This cross-sectional clinical investigation was approved by the Regional and Institutional Human Medical Biological Research Ethics Committee of Albert Szent-Györgyi Clinical Center at the University of Szeged. Informed consent was obtained from all participants in the study. Serum samples were collected at the first regular follow-up visits following the commencement of the clinical study, regardless of the patients' clinical status or treatment.

The PsA group comprised 46 patients (24 women and 22 men) who fulfilled the CASPAR classification criteria for PsA and who had been treated in the absence of any information as to their serologic status regarding antibody reactivities against citrullinated proteins. The basic demographic, clinical and laboratory characteristics on the PsA group are to be seen in Tables 1 and 2. The mean (±standard deviation; SD) age of these patients was 54.3 ± 11.9 years (range: 28–77 years). At the time of sampling collection, the mean disease activity score in 28 joints (DAS28) [38, 39] was 4.51 ± 1 (range: 2.08–6.81); the scores of 6 (13%) patients were ≤3.2 (low PsA activity), 27 (59%) had DAS28 scores between 3.3 and 5.1 (moderate PsA activity), and 13 (28%) had DAS28 scores >5.1 (high PsA activity). All patients had been previously or were currently treated with at least one type of disease-modifying antirheumatic drug (DMARD). The group was heterogeneous as regards the arthritis phenotypes. The Moll and Wright criteria [37] were used to classify the PsA patients into subgroups. Twenty-five patients (54%) had asymmetrical oligoarthritis, 20 (43%) had symmetrical polyarthritis, 8 (17%) had axial arthritis, and 2 (4%) had distal arthritis. There was only 1 patient with arthritis mutilans (2%). Fifteen PsA patients (33%) had distal interphalangeal (DIP) joint inflammation. All 46 patients also displayed psoriatic skin lesions. 

The basic characteristics on the 42 PsO patients are similarly given in Tables 1 and 2. Their mean age was 45.60 ± 15.72 years (range: 18–78 years), and the female : male ratio was 11 : 31 (26% versus 74%). The group consisted of 6 patients with a mild and 36 patients with a moderate-to-severe disease course. The assessment of the severity of the disease course was based on the previous and current antipsoriatic therapy: patients previously or currently treated with systemic (including biological) therapy or full-body phototherapy were considered to have “moderate-to-severe” PsO and the others to have “mild” PsO. At the time of serum sample collection, the mean psoriasis area and severity index (PASI) [40] score was 5.84 ± 6.75 (range: 0.00–34.20), but most patients were on concurrent systemic, biological, or phototherapy. None of the PsO patients had psoriatic joint involvement, as assessed by a trained rheumatologist. 

A randomly selected, self-stated healthy group of volunteers (*N* = 40) served as controls (none of them had ever exhibited psoriatic skin or joint symptoms). Their mean age was 45.05 ± 19.56 years (range: 16–82 years) and the female : male ratio was 20 : 20.

### 2.2. Determination of Anti-MCV IgG by ELISA

Anti-MCV IgG antibodies were analyzed by ELISA (ORG 548 anti-MCV; ORGENTEC Diagnostika GmbH, Mainz, Germany), with recombinant MCV as antigen. The analyses were conducted in accordance with the manufacturer's instructions. As recommended by the manufacturer, patients with anti-MCV titers higher than the 20 U/mL cut-off value were regarded as positive.

### 2.3. Statistical Analysis

The data on the anti-MCV-positive and negative patient groups were compared by means of the Fisher exact test. Since the data were not normally distributed, the correlations between anti-MCV positivity and the clinical features were determined through the use of nonparametric methods. Nonparametric methods were applied to assess overall group differences via pairwise comparisons (Mann-Whitney *U* tests) and Spearman's rank correlation coefficient. All statistical analyses were performed with the statistical program SPSS Windows (v15.0). *P*  values <0.05 were considered significant.

## 3. Results

### 3.1. Anti-MCV Titers Are Significantly Higher in PsA Than in PsO Patients and Nonpsoriatic Individuals

**Figure 1:**
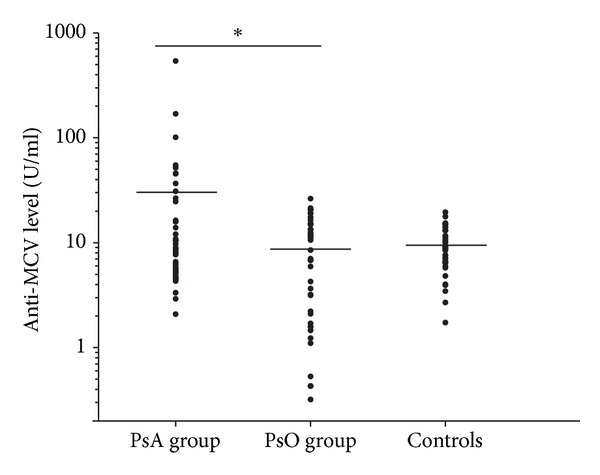
Anti-MCV titers are higher in PsA patients than in patients with psoriasis without arthritis and in healthy volunteers. The plots show the antibody levels of the investigated patients. The horizontal lines indicate the mean levels of anti-MCVs. The mean autoantibody level in the PsA group was 30.32 ± 82.14 U/mL as compared with 8.71 ± 7.41 U/mL in the PsO group and 9.50 ± 4.23 U/mL in the healthy control group. *The difference between the data on the PsA and PsO groups was statistically significant (*P* < 0.05).

As anti-MCV positivity is a characteristic hallmark of RA, we first investigated whether anti-MCVs are also associated with a different type of inflammatory joint disease, PsA. Our PsA patients exhibited significantly higher mean serum anti-MCV levels than those of the PsO patients (Figure 1): 30.32 ± 82.14 U/mL and 8.71 ± 7.41 U/mL, respectively. The mean antibody levels of the controls (9.50 ± 4.23 U/mL) and the PsO group did not differ significantly.

With the recommended cut-off value of 20 U/mL, 11 PsA patients (24%) and 3 PsO patients (8%) were found to be positive for anti-MCVs, whereas all of the controls were anti-MCV-negative. The differences between the PsA and PsO groups and between the PsA and control groups were statistically significant (*P* = 0.032 and *P* = 0.0009, resp.) but that between the PsO and control groups was not statistically significant (*P* = 0.0848).

### 3.2. A Higher Anti-MCV Titer in PsO Patients Is Associated with a More Severe Disease Course

**Figure 2:**
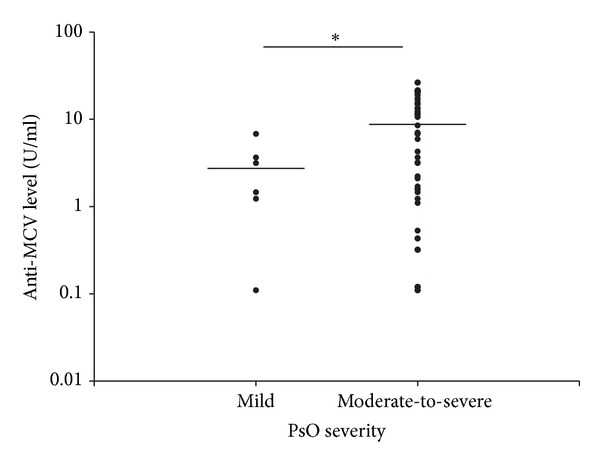
Anti-MCV titers are higher in patients with moderate-to-severe psoriasis than in mild psoriasis. The plots show the anti-MCV levels in moderate-to-severe (previously or currently treated with systemic or phototherapy) and mild (never received systemic or phototherapy) PsO patients. The horizontal lines indicate the mean levels of anti-MCVs. *The difference between the data on mild and the moderate-to-severe psoriasis PsO groups (2.73 ± 2.37 U/mL versus 9.73 ± 7.54 U/mL), respectively, was significant (*P* < 0.05).

**Figure 3:**
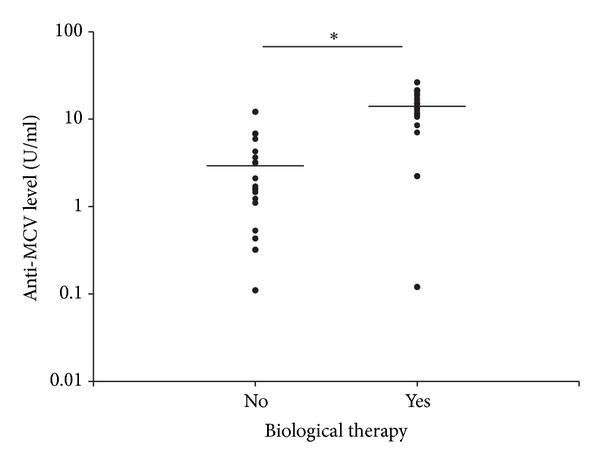
Anti-MCV titers are higher in severe PsO patients treated with biological therapy than in moderate-to-severe psoriasis patients not requiring biological therapy. The plots show the antibody levels of investigated patients. The horizontal lines indicate the mean levels of anti-MCVs. *The PsO patients not requiring biological therapy had significantly lower anti-MCV levels than those treated with biological therapy (3.01 ± 3.34 U/mL versus 14.01 ± 6.22 U/mL; *P* < 0.01).

Significantly higher anti-MCV titers were found in the PsO subgroup with a more severe disease course (9.73 ± 7.54 U/mL versus 2.73 ± 2.37 U/mL; *P* = 0.033) (Figure 2), although in both subgroups the mean titers were below the cut-off level proposed by the manufacturer. Furthermore, the moderate-to-severe PsO patients treated with biological therapy (Figure 3) demonstrated significantly higher anti-MCV levels than those of the moderate-to-severe patients who did not require biological therapy (14.01 ± 6.22 U/mL versus 3.01 ± 3.34 U/mL; *P* < 0.01) (both means were below the proposed cut-off level). On the other hand, the anti-MCV titers did not correlate significantly with the current disease activity (as determined by the PASI and DAS28 scores) at the time of serum sampling (data not shown). It seems, therefore, that the current psoriasis activity is not a critical determinant of the anti-MCV level in PsO. Rather, the PsO patients with the most severe disease course (requiring biological therapy) display the highest anti-MCV levels. A similar analysis was not feasible in the PsA group, which consisted almost entirely of severe cases who were receiving systemic DMARD or biological therapy. Similarly as in the PsO group, the current disease activity, as demonstrated by the DAS28 level, was not associated with higher anti-MCV levels in the PsA group either (*P* = 0.843; data not shown).

### 3.3. High Anti-MCV Titers in PsO Are Associated with an Early Onset of the Disease

**Figure 4:**
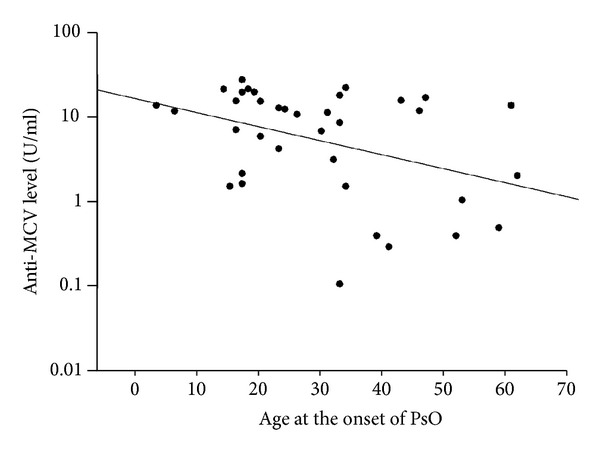
The anti-MCV levels demonstrate a significant inverse correlation with the age at the onset of the disease in the PsO patients. The plots represent the anti-MCV levels of the psoriasis patients and the age at the onset of psoriasis (*P* = 0.019).

As demonstrated in Figure 4, the anti-MCV levels proved to demonstrate a significant inverse correlation with the age at the onset of PsO (*P* = 0.019; patients who exhibited the early appearance of psoriatic skin symptoms usually presented with higher anti-MCV levels than those of the patients with a late disease onset). In the PsA group, however, no correlation was found between the age at PsA onset and the serum anti-MCV level (*P* = 0.096; data not shown).

### 3.4. The Presence of Tender Knee Joints and Nail Psoriasis Is Associated with Anti-MCV Positivity in PsA Patients

In order to identify clinical or laboratory features associated with high anti-MCV levels, we examined various parameters (Table 3), PsA patients were subdivided into anti-MCV-positive and negative groups, using the recommended cut-off value of 20 U/mL. A similar experiment, though seemingly reasonable, was not feasible in the PsO group, as the low number of anti-MCV-positives (*N* = 3) did not allow a meaningful statistical analysis in this group. 

**Figure 5:**
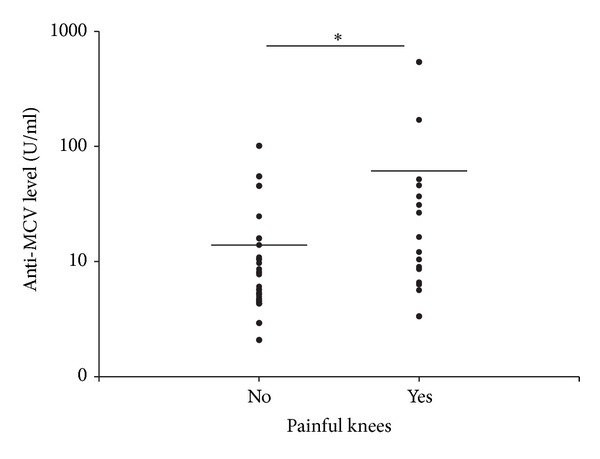
The anti-MCV levels in PsA patients with painful knees are significantly higher than in patients without painful knee. The plots show the antibody in PsA patients without pain of knees (labelled as “No”) and with pain of knees (labelled as “Yes”). The horizontal lines indicate the mean levels of anti-MCVs. *The difference between the anti-MCV levels in the two groups (13.87 ± 20.22 U/mL versus 61.18 ± 133.76 U/mL) was significant (*P* < 0.05).

Only two of these parameters proved to be correlated with high anti-MCV titers. The presence of painful knee joints was significantly more frequent in the anti-MCV-positive patients (63.64% versus 25.71%; *P* = 0.032), and a significantly higher mean anti-MCV titer was detected in the PsA subgroup with painful knees (61.18 ± 133.76 U/mL versus 13.87 ± 20.22 U/mL; *P* = 0.013) (Figure 5). However, there was no correlation, between the presence of painful knees and either the patient's age or the patient's body weight (data not shown). 

**Figure 6:**
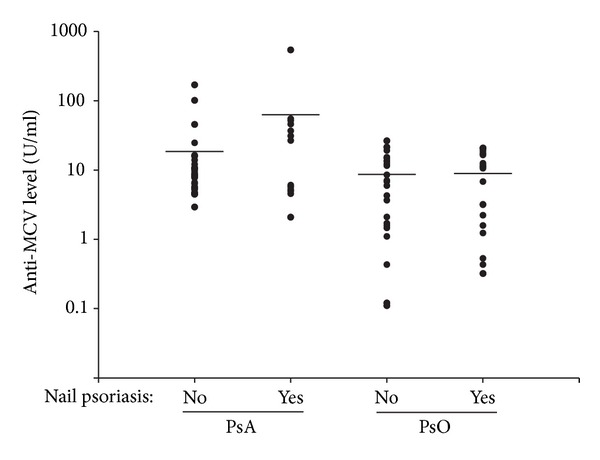
Anti-MCV levels of PsA and PsO patients with and without nail psoriasis. The plots represent the antibody levels of PsA and PsO patients with and without nail psoriasis. The horizontal lines indicate the mean levels of anti-MCVs. The differences between the groups were not significant (*P* = 0.305).

The second clinical feature that was significantly more frequent in the anti-MCV-positive PsA group was the presence of nail psoriasis (63.64% and 17.14% in the patients with and without psoriatic nail symptoms, resp.; *P* = 0.006). However, when the PsA and PsO patients were subdivided on the basis of the presence of nail symptoms, although a clear tendency was observed toward a higher anti-MCV level in those with nail symptoms, the difference was not statistically significant (*P* = 0.305) (Figure 6). 

## 4. Discussion

This study has demonstrated significantly higher anti-MCV titers in PsA patients than in PsO patients or in healthy controls. The mean autoantibody level in the PsA group was 30.3 U/mL, as compared with 8.7 U/mL in the PsO group and 9.5 U/mL in the control group. The serum anti-MCV concentrations, although clearly higher in a subset of PsA patients, were markedly lower than the values of several hundred-to-thousand U/mL reported previously in RA patients [19]. Similar to our observations, modestly elevated anti-MCV titers have been reported in a subpopulation of PsA [18, 41] and in ankylosing spondylitis patients [42]. In our study cohort, 24% (11 out of 46) of the PsA patients were found to be anti-MCV-positive. To the best of our knowledge, anti-MCV levels in PsA have been reported only twice previously, and the results were not in full concordance, the prevalence of anti-MCV positivity in PsA ranging from 3.6% [41] to 15.2% [18]. The cause of the even higher anti-MCV positivity rate in our study population cannot be fully explained. The presence of anti-MCVs was earlier reported to correlate significantly with the disease activity in RA [19, 20]. In our study, the disease activity (as determined by the PASI and the DAS28 scores) was not associated with elevated anti-MCV titers (data not shown), though almost all of our patients were actively treated with DMARDs or biologicals at the time of sample collection. In the anti-MCV-positive patients separately, the average number of swollen joints was relatively low (3.45 ± 2.94) and the disease activity reflected by the DAS28 score was moderate (4.49 ± 0.98).

Nail psoriasis and tender knee joints were observed significantly more frequently in the anti-MCV-positive PsA patients than in the anti-MCV seronegatives (64% versus 17%). It has recently been recognized that distal DIP joint disease in PsA is associated with diffuse inflammation that envelops the nail root and adjacent bone [43]. Thus, nail matrix inflammation and therefore psoriatic nail changes result from PsA enthesitis, and consequently nail psoriasis reflects DIP joint enthesitis. More recently, it has been demonstrated that nail involvement in psoriasis is directly correlated with systemic enthesitis, as the enthesopathy scores are significantly higher in PsA patients with nail disease than in those without it [44]. Overall, the association of anti-MCV positivity with psoriatic nail symptoms in our study population may indicate that a high anti-MCV level is a marker of systemic enthesitis in PsA. However, we could not confirm this hypothesis through a direct comparison of the enthesopathy scores with the anti-MCV titers, as the presence of subclinical enthesitis was not recorded at the time of sample collection in our study. Whether the increased number of PsA patients with tender knee joints within the group of anti-MCV seropositives is an epiphenomenon, or reflects a clear pathogenetic association, requires further investigations. 

Although the mean anti-MCV titer of the PsO patients did not differ from that for the healthy controls, when the PsO patients were divided into moderate-to-severe and mild groups, higher anti-MCV antibody titers proved to be significantly associated with a more severe disease course. Furthermore, those treated with biological therapy had significantly higher anti-MCV levels than the moderate-to-severe psoriasis patients who did not require biological therapy. Although the mean anti-MCV titers were below the proposed cut-off level, these findings imply that, within the group of PsO patients, higher anti-MCV levels may distinguish those patients with a more severe disease course. Whether these patients have significant subclinical joint involvement potentially detectable with highly sensitive imaging methods is unclear. Furthermore, we cannot exclude the possibility that these patients will eventually develop clinically evident PsA. 

As regards the role of (biological) therapy in ACPA levels, some studies have reported significantly decreased serum RF and anti-CCP levels in RA patients in response to 6–12 months of TNF inhibitor therapy [45–47]. Nicaise Roland et al. observed significantly decreased anti-MCV levels after 18–24 months of anti-TNF treatment in RA [48]. Several other studies, however, did not detect marked changes in anti-CCP levels after 22, 30, or 54 weeks of infliximab treatment in RA [49–51]. We are not aware of any literature report of increased anti-MCV (or ACPA) levels in patients treated with biological therapy. Even though a definite conclusion cannot be drawn from these findings, these studies indicate that anti-CCPs are most probably not influenced (or at least are not increased) by anti-TNF-*α* therapy and are a relatively stable hallmark of RA. In view of the generally low (lower than the manufacturer-recommended cut-off value) anti-MCV titers in our PsO population, it seems unlikely that biological treatment would significantly modify the anti-MCV levels. If therapy had an effect on anti-MCV levels, this would result in decreasing titers in patients on biological therapy. In our opinion, therefore, the higher antibody titers in the group of PsO patients treated with anti-TNF therapy are not directly related to the treatment, but rather to the underlying severe disease course leading to the use of biologicals. Indeed, anti-MCV positivity is associated with a more severe disease course and a poor radiographic prognosis in patients with early RA [19]. 

The idea that a more severe disease course in PsO is associated with higher anti-MCV level was supported by the finding that the anti-MCV levels displayed a significant inverse correlation with the age at the onset of psoriatic skin symptoms. It is well known that patients with early-onset PsO usually have a more significant genetic background (HLA-cw6) and, among others, develop PsA more frequently [52, 53]. Early-onset PsO is frequently associated with a more severe disease course, and patients with early-onset PsO are therefore potentially more likely to suffer from PsA and more severe skin symptoms. 

In conclusion, our study suggests that anti-MCVs, apart from being biomarkers of early RA, can also be used to differentiate a subset of PsO patients. As the differentiation of early and mild forms of PsA can pose a significant challenge in some cases, the detection of anti-MCV positivity can aid the diagnosis of PsA, especially in patients with psoriatic nail symptoms and tender knee joints. Furthermore, high levels of anti-MCVs in PsO patients without clinically manifest arthritis may distinguish patients who are more likely to experience a severe disease course and potentially require biological therapy. However, this study needs to be extended to large groups of both PsA and PsO patients in order to confirm these associations.

## Figures and Tables

**Table 1:** Basic demographic and clinical characteristics of psoriatic arthritis (PsA) and psoriasis (PsO) groups.

Variable	PsA group (*N* = 46)	PsO group (*N* = 42)
Male : female ratio	24 : 22	31 : 11
Age (mean ± SD; years)	54.35 ± 11.87	45.60 ± 15.72
BMI (mean ± SD; kg/m^2^)	29.38 ± 6.41	28.86 ± 9.80
Current smokers (%)	20	12
Age at diagnosis of PsO (mean ± SD; years)	38.91 ± 14.47	28.84 ± 15.82
Age at diagnosis of PsA (mean ± SD; years)	45.26 ± 13.80	—
Disease course severity (mild : moderate to severe)^•^	0 : 46	6 : 36
Psoriasis guttata (%)	4	20
Arthritis mutilans (%)	2	—
Axial arthritis (%)	17	—
Distal arthritis (%)	4	—
Asymmetrical oligoarthritis (%)	54	—
Symmetrical polyarthritis (%)	43	—
Therapy		
Received MTX therapy (%)	85	57
Received systemic steroid treatment (%)	13	2
Received 311 nm NB-UVB therapy (%)	7	38
Received PUVA therapy (%)	2	31
Received biological therapy (%)	13	52

PsO: psoriasis vulgaris, PsA: psoriatic arthritis, BMI: body mass index, MTX: methotrexate, PUVA: psoralen + ultraviolet A, 311 nm NB-UVB: 311-nanometer narrow-band ultraviolet B. Symmetrical arthritis: bilateral arthritis with a frequency of >50%.

^•^Patients previously or currently treated with DMARDs, systemic therapy or full-body phototherapy were regarded as “moderate-to-severe” patients, whereas the others were considered to exhibit a “mild” disease course.

**Table 2:** Clinical and laboratory characteristics of patients in the psoriatic arthritis (PsA) and psoriasis vulgaris (PsO) groups.

Variable	PsA group (*N* = 46)	PsO group (*N* = 42)
Anti-MCV positivity (%)	24	8
Level of anti-MCV (mean ± SD; U/mL)	30.32 ± 82.14	8.71 ± 7.41
ANA positivity (%)^†^	38	Not measured
RF positivity (>9 U/mL; %)^†^	11	Not measured
Active psoriatic lesions in the skin	100	95
PASI score (mean ± SD)	—	5.84 ± 6.75
Nail psoriasis (%)	28	43
Scalp psoriasis (%)	72	57
Plaques on the face (%)	11	14
Plaques on the upper limbs (%)	61	71
Plaques on the trunk (%)	30	48
Plaques on the perineum (%)	15	7
Plaques on the lower limbs (%)	59	88
Arthritic features		
DAS28 score (mean ± SD)	4.51 ± 1.00	—
DIP involvement (%)	33	—
Erosion (%)	24	—
Tender joint count (mean ± SD)	9.78 ± 5.90	—
Back (%)	48	—
Shoulders (%)	37	—
Elbows (%)	15	—
Wrists (%)	46	—
Hands (%)	67	—
Hips (%)	17	—
Knees (%)	35	—
Feet (%)	61	—
Swollen joint count (mean ± SD)	2.67 ± 3.19	—
Swollen shoulder (%)	0	—
Swollen elbow (%)	2	—
Swollen wrist (%)	9	—
Hand joints (%)	43	—
Swollen knee (%)	11	—
Feet joints (%)	26	—

Anti-MCVs: antibodies against mutated citrullinated vimentin, ANA: anti-nuclear antibody, RF: rheumatoid factor, DIP: distal interphallangeal, PASI: psoriasis area and severity index, DAS28: disease activity score.

**Table 3:** Comparisons of clinical findings in anti-MCV-positive and anti-MCV-negative PsA patients.

Variable	Anti-MCV-positive PsA patients (*N = *11)	Anti-MCV-negative PsA patients (*N = *35)
Sex ratio (male : female)	3 : 8	21 : 14
Age (mean ± SD; years)	57.91 ± 9.26	53.23 ± 12.48
Current smoker (%)	9	23
Age at diagnosis of PsO (mean ± SD; years)	44.27 ± 13.73	37.23 ± 14.48
Age at diagnosis of PsA (mean ± SD; years)	46.55 ± 15.78	44.86 ± 13.34
PsA severity (mild : moderate to severe)	0 : 11	0 : 35
Psoriasis guttata (%)	9	3
Arthritis mutilans	9	0
Axial type (%)	27	14
Distal type (%)	9	3
Asymmetrical oligoarthritis (%)	64	51
Symmetrical polyarthritis (%)	36	46
Therapy		
Received local steroid treatment (%)	73	83
Received sulfasalazine (%)	27	20
Received systemic steroid treatment (%)	27	9
Received 311 nm NB-UVB therapy (%)	18	3
Received PUVA therapy (%)	0	3
Received MTX therapy (%)	73	89
Received biological therapy (%)	18	11
DIP involvement (%)	18	37
Erosion (%)	18	26
Level of anti-MCV (mean ± SD; U/mL)	**102.41 ± 150.99**	7.67 ± 3.77*
ANA positivity (%)^†^	50	33
RF positivity (%)^†^	0	14
DAS28 score (mean ± SD)	4.49 ± 0.98	4.52 ± 1.02
Psoriatic skin lesions		
*** ***Nail psoriasis (%)	**64**	17*
Scalp psoriasis (%)	64	74
Plaques on the face (%)	9	11
Plaques on the upper limbs (%)	55	63
Plaques on the trunk (%)	36	29
Plaques on the perineum (%)	9	17
Plaques on the lower limbs (%)	55	60
Arthritic features		
Tender joint count (mean ± SD)	7.27 ± 3.58	10.57 ± 6.29
Back (%)	36	51
Shoulders (%)	36	37
Elbows (%)	18	14
Wrists (%)	45	46
Hands (%)	45	74
Hips (%)	0	23
** ** Knees (%)	**64**	26*
Feet (%)	73	57
Swollen joint count (mean ± SD)	3.45 ± 2.94	2.43 ± 3.27
Swollen shoulder (%)	0	0
Swollen elbow (%)	9	0
Swollen wrist (%)	18	6
Hand joints (%)	45	40
Swollen knee (%)	27	6
Feet joints (%)	18	29

PsO: psoriasis vulgaris, PsA: psoriatic arthritis, anti-MCVs: antibodies against mutated citrullinated vimentin, BMI: body mass index, PASI: psoriasis area and severity index, DAS28: disease activity score, DIP: distal interphalangeal, HLA B27: human leukocyte antigen B27, ANA: anti-nuclear antibodies, RF: rheumatoid factor, ESR: erythrocyte sedimentation rate, CRP: C-reactive protein, MTX: methotrexate, PUVA: psoralen + ultraviolet A, nm: nanometer. Symmetrical arthritis: bilateral arthritis with a frequency of >50%. ^†^The current values in these cases related to at least in 60%. For the other values, the data were complete: 100%. *There were significant differences between the anti-MCV-positive and negative groups (*P* < 0.05).
